# The Cognitive and Behavioural Profile of Amyotrophic Lateral Sclerosis: Application of the Consensus Criteria

**DOI:** 10.3233/BEN-2012-110202

**Published:** 2013

**Authors:** Monica Consonni, Sandro Iannaccone, Chiara Cerami, Paola Frasson, Marco Lacerenza, Christian Lunetta, Massimo Corbo, Stefano F. Cappa

**Affiliations:** ^1^Department of Clinical Neurosciences and Division of NeuroscienceSan Raffaele Scientific InstituteMilanItaly; ^2^Vita-Salute UniversityVia OlgettinaMilanItaly; ^3^NEuroMuscular Omnicentre–Fondazione Serena OnlusNiguarda Ca’ Granda HospitalMilanItaly

**Keywords:** Amyotrophic Lateral Sclerosis, Lower Motor Neuron Disease, cognitive impairment, behavioural impairment, dysexecutive syndrome, motor disability

## Abstract

**Objective:** The study aims to assess the spectrum of cognitive and behavioural disorders in patients affected by Amyotrophic Lateral Sclerosis (ALS) according to the recent consensus criteria [9]. The study also intends to assess the impact of physical disability on cognitive and behavioural abnormalities.

**Methods:** Detailed neurological, neuropsychological and neurobehavioral evaluations were administered to 23 ALS patients, 11 Lower Motor Neuron Disease (LMND) patients and 39 healthy controls. Strong et al.’s criteria [9] were applied to diagnose the presence of cognitive/behavioural impairment. Clinical and neuropsychological scores were used for group comparisons and correlation analyses.

**Results:** In comparison with LMND and controls, a subgroup of ALS patients (∼30%) manifested executive dysfunction, which was severe enough to classify them as cognitively impaired. Action naming difficulties and short-term memory deficits were also observed. Aspontaneity, disorganization and mental rigidity reached clinical relevance in 20% of ALS patients. A small percentage of ALS patients (13%) also had comorbid dementia. The cognitive or behavioural status was not related to the clinical features of ALS.

**Conclusion:** The use of consensus criteria for cognitive and behavioural impairment and the comparison with the LMND group proved useful in defining the spectrum of non-motor manifestations of ALS.

## 1. Introduction

Amyotrophic Lateral Sclerosis (ALS) is a multisystem disorder, in which the core pathology is the degeneration of motor neurons, giving rise to progressive and diffuse muscle wasting, weakness and spasticity. In addition, a spectrum of cognitive and/or behavioural dysfunctions constitutes the non-motor manifestations of ALS.

Although a subset of patients, ranging from 1 to 40% [1–4] fulfils the criteria for dementia, the cognitive impairment in ALS is most commonly characterised by a mild or moderate dysfunction in the domain of executive functions (e.g. word generation, problem solving, attentional control and reasoning). Memory and language deficits have been less frequently reported [5]. The estimated prevalence of cognitive dysfunction ranges from 10% to 75% [1,3,6–8]. This wide variation can be attributed to the selection of patients and

ISSN 0953-4180/13/$27.50 © 2013 – IOS Press and the authors. All rights reserved the methods used for the diagnosis. Recently, a consensus on diagnostic criteria of cognitive impairment in ALS (ALSci) has been reached. ALSci is defined as when the patient's performance is below the fifth percentile on at least two distinct standardized neuropsychological tests evaluating executive functions [9].

Few studies addressed the behavioural aspects of the disorder. The concept that behavioural impairment in ALS (ALSbi) is rare is no longer supported by clinical evidence [10]. Apathy, disinhibition and poor social monitoring are frequently reported in a subgroup of ALS patients meeting criteria for frontotemporal dementia (FTD) [11], with a prevalence from 22% to 52% [2,12]. In addition, mild to moderate behavioural impairments have also been documented in non-FTD ALS patients [13]. Apathy [14,15], self-centeredness and irritability [10] seem to be the most frequent behavioural changes of ALS. Whether these are symptoms of frontal lobe damage or emotional reactions to the progressive physical disabilities is still a matter of debate.

The present study aims to assess the spectrum of cognitive and behavioural dysfunctions, defined according to published consensus criteria [9,11] in a consecutive cohort of ALS patients without a diagnosis of dementia antedating the ALS diagnosis. It also aims to assess the presence of differences in clinical, neuropsychological and neurobehavioral features between ALSci/ALSbi and cognitively-normal ALS patients. To this end, we applied an extensive battery of neuropsychological tests assessing executive function as well as other cognitive domains. This allowed us to evaluate the specificity of executive dysfunction, and its potential impact on other cognitive domains. In addition, the study aims to distinguish cognitive and behavioural changes that may be due to extra-motor cortical involvement in ALS from those that can be a consequence of physical disability due to motor neuron involvement and emotional reactions to motor disability. For this purpose, a group of Lower Motor Neuron Disease (LMND) patients was recruited and compared to the ALS group, and correlations between cognitive performances and clinical features of patients were sought.

## 2. Methods

### 2.1. Participants

#### 2.1.1. Patients

We consecutively recruited thirty-nine motor neuron disease outpatients of the Neurological Unit, Department of Clinical Neuroscience, San Raffaele Turro Hospital and NEuroMuscular Omnicentre in Milan. Informed consent was obtained from all subjects, and the local ethical committee approved the investigation. Inclusion criteria were history and neurological examination findings consistent with ALS or LMND, supplemented by confirmatory electromyographic findings. Since the occurrence of ALS in FTD patients is well-known [16], we excluded patients with a diagnosis of dementia who later developed signs of motor neuron impairment, in order to define the spectrum of cognitive and/or behavioural impairments in patients presenting with motor disabilities. In addition, individuals with a history of head trauma, depression or other psychiatric diseases and those with evidence of other pathologies on MRI scans were excluded. Patients with a family history of neurodegenerative disease were eligible for inclusion. Upper motor neuron involvement was confirmed by clinical evaluation, MRI scans and motor-evoked potentials. Five motor neuron disease patients did not fulfil inclusion criteria and were excluded (2 primary lateral sclerosis, 1 ALS with sub-cortical tumour, 1 ALS patient with a previous diagnosis of primary progressive aphasia, 1 progressive muscular atrophy-like patient with familial hyperCKemia).

We included 23 patients meeting the international criteria for clinical diagnosis of probable or definite sporadic ALS [17,18] and 11 patients (4 female), affected by lower motor neuron diseases (LMND) as the control disease group. The LMND group consisted of 8 patients with progressive muscular atrophy (PMA), 1 PMA with diabetes, 1 monomelic amyotrophy, and 1 spinal muscular atrophy. None of the patients was receiving assisted ventilation.

#### 2.1.2. Controls

Thirty-nine healthy subjects (22 females) were enrolled. Controls with concomitant conditions that might affect test performance (e.g. stroke, depression and other neurological diseases) were excluded before sampling.

### 2.2. Clinical assessment

#### 2.2.1. Neurological evaluation

All patients underwent a structured clinical interview. The severity of clinical disability was assessed with the ALS-Norris Scale (ALS-N) [19]. The degree of bulbar and spinal impairment was evaluated with the first two and last two items of the Amyotrophic Lateral Sclerosis Severity Scale (ALSSS) [20]. For all patients, disease duration (in months) and disease onset type (bulbar or spinal) were reported. Depression was evaluated with the Hamilton Depression Rating Scale (HDRS) [21]. Diagnosis of FTD was based on the Neary criteria [11].

### 2.2.2. *Neuropsychological assessment*

A standard neuropsychological test battery was administered to all participants by evaluators (M.C. and P.F.), blinded to diagnostic category of patients. Multiple areas of cognition were assessed using the Mini Mental State Examination (MMSE) [22], Digit Span Forward [23,24], the Rey Auditory Verbal Learning test (RAVL) [25], Raven Coloured Progressive Matrices [26], letter (P-F-L) and category (animals-fruits-cars) fluency tests [27]. All subjects were then invited to participate in a detailed neuropsychological and neurobehavioral assessment for research purposes. The following tests were added: the Stroop Interference Test [28], the Wisconsin Card Sorting Test (WCST) [29], the Cognitive Estimation Task (CET) [30], Digit span backwards [23] and the object and action naming subtests of B.A.D.A. [31].

Age- and sex-corrected norms were used to generate standardized scores [32]. Standardized scores range from 0 (pathological performance = performance below 5% of the normal distribution) to 4 (performance above the mean of the normal distribution). To control for individual variations in motor speed, the mean fluency indexes were calculated [33] and the reading time difference between colour and word-colour-interference conditions of the Stroop test was considered. Patients with arm weakness were assisted by an examiner who moved the cards as instructed during the WCST.

### 2.2.3. *Neurobehavioral assessment*

The behavioural assessment was performed through patient observation and a structured interview with an adult family member of each patient. For this purpose, we used the Italian version of the Frontal Behavioural Inventory (FBI) [34,35]. Items of the original interview schedule that might be biased because of physical disability were excluded on a case-by-case basis.

## 2.3. *Defining cognitive and behavioural abnormalities*

In the first step of the study, we identified the prevalence of dementia, cognitive and behavioural impairments in ALS and LMND patients. Means and 95% confidence interval were calculated for all neuropsychological measurements. Diagnosis of cognitive (ALSci) and behavioural (ALSbi) impairment in ALS was based on recently published consensus criteria [9]. Patients with cognitive performance at or below the fifth percentile on at least two standardized neuropsychological tests sensitive to executive functioning were diagnosed with ALSci. The impairment was not accounted for by the patient's premorbid intellectual level, depression, bulbar dysfunction (dysarthria) and motor weakness. According to Strong and colleagues [9], the diagnosis of behavioural impairment requires that individuals meet at least 2 non-overlapping supportive diagnostic features from either the Neary criteria [11] and/or Hodges' criteria [36]. Patients may meet the criteria for both ALSci and ALSbi concurrently, and they can change diagnostic categories with disease progression.

Demographic and clinical data of unimpaired ALS, ALSci/ALSbi, LMND and control groups were compared using the Kruskal-Wallis H test and the Mann-Whitney U test.

The mean number of pathological performances (i.e.: scores below the fifth percentile compared to norms) within each subject group for executive function, memory and language domains was calculated, and the Kruskal-Wallis H test and the Mann-Whitney U test were used to test group differences. The Bonferroni correction for multiple comparisons was also applied.

To further qualify the pattern of cognitive impairment, we then compared each neuropsychological test score of ALSci/ALSbi and cognitively-normal ALS groups with those of LMND and control groups. The analyses were conducted using the Kruskal-Wallis H test. If significant differences or trends were detected, post hoc analyses were performed with the Mann-Whitney U test. Hedge's g effect size was calculated for ALS and LMND groups. The Bonferroni correction for multiple comparisons was applied.

To identify the pattern of behavioural impairment, the Kruskal-Wallis H and the Fisher's exact test were used, respectively, to assess differences in FBI scores and in the number of behavioural anomalies (as revealed with FBI) between ALSci/ALSbi, unimpaired ALS, and LMND patients, with Bonferroni correction for multiple comparisons.

To test if motor impairment and disease duration were related to cognitive deficits or behavioural abnormalities, correlation analysis was performed between uncorrected neuropsychological scores and clinical features of ALS and LMND patients.

Table 1
Clinical data of ALS and LMND patients

| | ALS Group | | | | | | | | Sub-group comparisons |
|---|---|---|---|---|---|---|---|---|---|
| | Unimpaired ALS (11 patients) | | ALSci/ALSbi (9 patients) | | ALS/Dementia (3 patients) | | LMND (11 patients) | | |
| Clinical Data | Mean | C.I. | Mean | C.I. | Mean | Min-Max | Mean | C.I. | |
| Age | *58.00* | 51–65 | *63.11* | 53–73 | *65.33* | 53–73 | *59.27* | 52–67 | N.S. |
| Education (years) | *10.55* | 7–14 | *7.67* | 5–10 | *7.00* | 3–13 | *9.36* | 6–12 | N.S. |
| Male/female ratio | *7/4* | – | *3/6* | – | *1/2* | – | *7/4* | – | N.S. |
| Disease Duration (months) | *15.18* | 4–26 | *16.22* | 7–25 | *10.67* | 8–14 | *66.82* | 34–99 | LMND > unimpaired ALS**† LMND > ALSci/ALSbi*† |
| ALS-N Score | *85.18* | 78–92 | *73.22* | 58–88 | *73.33* | 39–91 | *82.09* | 73–90 | N.S. |
| ALSSS Bulbar score | *17.09* | 15–19 | *18.78* | 17–20 | *13.00* | 5–19 | *19.18* | 18–20 | N.S. |
| ALSSS Spinal score | *16.64* | 15–18 | *16.22* | 7–25 | *15.67* | 10–20 | *15.18* | 13–18 | N.S. |
| Bulbar/Spinal Onset | *3/8* | – | *1/8* | – | *2/1* | – | *0/9* | – | N.S. |
| HDRS | *7.18* | 3–12 | *4.78* | 1–8 | *6.6* | 0–10 | *3.63* | 1–6 | N.S. |
| FBI | *2.20* | 0–4 | *5.50* | 1–10 | *8.00* | 0–13 | *2.78* | 0–5 | N.S. |

Mean and confidence interval (C.I.) and the significant results of post-hoc analysis (Mann-Whitney U test) are reported. $^{*}p < 0.05$; $^{**}p < 0.01$; $^{\dagger}$ = Bonferroni correction for multiple comparisons ($p < 0.025$); N.S. = no significant difference. Legend: ALS-N = Amyotrophic Lateral Sclerosis – Norris Scale; ALSS = Amyotrophic Lateral Sclerosis Severity Scale; FBI = Frontal behavioural Inventory; HDRS = Hamilton Depression Rating Scale.

The results for demented ALS patients are reported in Tables 1 and 2. The demented ALS group were excluded from the analysis because of the small size of the sample and to avoid circularity (since neuropsychological test scores were used as dependent variables).

## 3. Results

### *3.1. Patient subgroups*

Based on clinical evaluations performed by two expert neurologists (S.I. and C.C.), 3 ALS patients were found to meet the criteria for dementia. Two patients met Neary et al.'s research criteria for the behavioural variant of FTD [11], with behavioural anomalies and dysexecutive syndrome. The first FTD case was a 53 year old woman with a positive family history of ALS/FTD, the other was a 70 year old man with sporadic ALS and long-term memory deficit. One additional patient (a 73 year old woman) had serious bulbar impairment (dysarthria and dysphagia) and diagnosis of probable Alzheimer Disease on the basis of NINCDS/ADRDA criteria [37]; she was disoriented in time and had severe deficits in memory, naming and auditory verbal comprehension.

According to the new consensus criteria [9], 11 ALS patients were cognitively and behaviourally normal, 7 (4 females) fulfilled criteria for ALSci, 1 (female) for ALSbi and 1 (female) had both cognitive and behavioural impairments (Fig. 1). Of the 11 LMND patients, none satisfied criteria for cognitive impairment, whereas 1 patient with PMA (female) satisfied criteria for behavioural impairment. Among the 39 control subjects who performed the standard evaluation, none satisfied criteria for cognitive impairment.

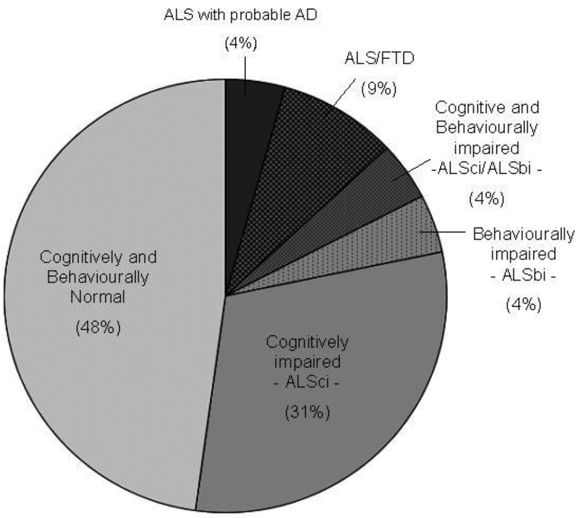

Fig. 1. Prevalence of dementia syndrome and cognitive and behavioural impairments in ALS. The distribution of dementia syndrome and cognitive and behavioural impairments in 23 patients with amyotrophic lateral sclerosis who, at the time of first evaluation, did not have previous diagnosis of cognitive impairment. Legend: AD = Alzheimer disease; ALS/FTD = ALS with comorbid FTD.

### *3.2. Demographic and clinical findings of patient subgroups*

Demographic and clinical data are reported in Table 1. No differences were found with respect to gender, age and education between unimpaired ALS,

Table 2

Neuropsychological performances of healthy controls, LMND patients and ALS patients

| | Control | | LMND | | Unimpaired ALS (uALS) | | ALSci/ ALSbi | | Demented ALS | Hodge's g (effect size) | | | | Group comparisons |
|---|---|---|---|---|---|---|---|---|---|---|---|---|---|---|
| | | | | | | | | | | LMND | uALS | ALSci/ALSbi | ALS Dementia | |
| *Measures* | *n.* Mean (C.I.) | *n.* | Mean (C.I.) | *n.* | Mean (C.I.) | n. | Mean (C.I.) | n. | Mean (min-max) | | | | | |
| *Demographical data* | | | | | | | | | | | | | | |
| Age | 39 61.4 (58–64) | 11 | 59.3 (52–67) | 11 | 58.0 (51–65) | 9 | 63.1 (53–73) | 3 | 65.3 (53–73) | [0.236] | [0.359] | [−0.166] | [−0.414] | N.S. |
| Education | 39 10.3 (9–12) | 11 | 9.36 (6–12) | 11 | 10.5 (7–14) | 9 | 7.7 (5–11) | 3 | 7.0 (3–13) | [0.265] | [−0.042] | [0.733] | [*0.897*] | N.S. |
| *Global efficiency* | | | | | | | | | | | | | | |
| MMSE | 39 28.2 (28–29) | 11 | 27.0 (25–29) | 11 | 27.5 (26–28) | 8 | 25.1 (23–27) | 2 | 22.7 (14–28) | [0.677] | [0.469] | [*1.702*] | [*2.763*] | * Control > ALsci/ALSbi $p = 0.002$<br>uALS > ALsci/ALSbi $p = 0.029$ |
| *Executive Functions* | | | | | | | | | | | | | | |
| Verbal phonemic fluency | 38 37.1 (33–41) | 11 | 29.5 (23–36) | 11 | 28.7 (21–37) | 9 | 25.3 (14–37) | 2 | 12.5 (12–13) | [0.645] | [0.698] | [*0.929*] | [*2.069*] | Control > ALsci/ALSbi $p = 0.038$<br>Control > uALS p=.038<br>Control > LMND $p = 0.040$ |
| Verbal category fluency | 38 44.4 (41–48) | 11 | 41.0 (33–49) | 11 | 41.0 (32–50) | 9 | 32.5 (23–42) | 2 | 24 (23–25) | [0,315] | [0.307] | [*1.069*] | [*1.936*] | * Control > ALsci/ALSbi $p = 0.002$ |
| Phonemic fluency index | 27 5.4 (4–7) | 10 | 6.5 (5–8) | 9 | 6.3 (4–8) | 8 | 14.8 (2–29) | 2 | 15.6 (13–18) | [0.406] | [−0.293] | [*−1,092*] | [*−3.304*] | N.S. |
| Category fluency index | 27 4.1 (3–5) | 10 | 4.6 (3–6) | 9 | 3.6 (3–4) | 8 | 6.6 (4–9) | 2 | 7.0 (7–7) | [−0.300] | [0.306] | [*−1.168*] | [*−1.734*] | * Control < ALsci/ALSbi $p = 0.013$<br>* uALS < ALsci/ALSbi $p = 0.016$ |
| WCST. global score | 16 66.2 (45–88) | 9 | 92.5 (72–113) | 9 | 57.2 (23–92) | 9 | 103.2 (80–127) | 1 | 118 | [−0.702] | [0.209] | [*−0.906*] | – | Control < ALsci/ALSbi $p = 0.018$<br>* uALS < ALsci/ALSbi $p = 0.014$ |
| WCST Perseverations | 16 22.7 (14–32) | 9 | 42 (15–68) | 9 | 17.2 (6–29) | 9 | 46 (26–66) | 1 | 118 | [−0.769] | [0.329] | [*−1.095*] | – | Control < ALsci/ALSbi $p = 0.029$<br>* uALS < ALsci/ALSbi $p = 0.012$<br>uALS < LMND $p = 0.049$ |
| WCST Errors | 16 17.4 (12–23) | 9 | 22.9 (12–33) | 9 | 18.2 (3–34) | 9 | 24.0 (9–39) | 1 | 0 | [−0.440] | [−0.051] | [−0.437] | – | N.S. |
| WCST Failures | 16 1.6 (1–2) | 9 | 1.7 (0–3) | 9 | 1.4 (0–3) | 9 | 2.0 (0–4) | 1 | 0 | [−0.025] | [0.120] | [−0.190] | – | N.S. |
| Stroop Interference (sec) | 21 56.5 (33–80) | 7 | 61.0 (30–92) | 9 | 50 (22–77) | 7 | 99.9 (39–161) | 2 | 132.2 (89–176) | [−0.089] | [0.132] | [−0.755] | [*−1.385*] | N.S. |
| No. Errors in Stroop test | 21 2.6 (0–5) | 7 | 2.7 (0–5) | 9 | 2.5 (0–7) | 7 | 5.6 (0–10) | 2 | 13 (0–26) | [−0.016] | [0.011] | [−0.529] | [*−1.493*] | Control < ALsci/ALSbi $p = 0.029$ |
| Raven Coloured Matrices | 39 29.8 (28–31) | 11 | 27.1 (24–30) | 11 | 28.8 (25–33) | 9 | 23.8 (19–29) | 3 | 15 (7–20) | [0.575] | [0.191] | [*1.194*] | [*3.015*] | * Control > ALsci/ALSbi $p = 0.007$ |
| Cognitive estimates (CET) | 20 13.0 (11–15) | 9 | 13.6 (12–15) | 8 | 12.0 (9–14) | 9 | 19.4 (16–23) | 2 | 18.5 (14–23) | [−0.160] | [0.284] | [*−1.641*] | [*−1.411*] | * Control < ALsci/ALSbi $p = 0.001$<br>* uALS < ALsci/ALSbi $p = 0.002$<br>* LMND < ALsci/ALSbi $p = 0.001$ |
| Bizarreness (CET) | 20 2.5 (2–3) | 9 | 3 (2–4) | 8 | 2.6 (1–4) | 9 | 7.2 (4–10) | 2 | 2.5 (1–4) | [−0.241] | [−0.042] | [*−1.700*] | [0.024] | * Control < ALsci/ALSbi $p = .001$<br>* uALS < ALsci/ALSbi $p = 0.001$<br>* LMND < ALsci/ALSbi $p = 0.001$ |
| Digit Span Backward | 21 4.4 (4–5) | 10 | 4.0 (3–5) | 9 | 4 (3–5) | 8 | 3.3 (2–4) | 2 | 1.0 (0–2) | [0.392] | [0.373] | [*0.896*] | [*1.516*] | N.S. |
| *Memory* | | | | | | | | | | | | | | |
| Digit Span Forward | 21 6.1 (6–7) | 10 | 5.1 (4–6) | 10 | 5.3 (5–6) | 9 | 4.8 (4–5) | 2 | 5.0 (4–6) | [*0.896*] | [0.730] | [*1.171*] | [*0.887*] | * Control > ALsci/ALSbi $p = 0.006$<br>Control > LMND $p = 0.023$ |
| Short-term recall (Rey list) | 39 48.3 (45–52) | 11 | 42.0 (35–49) | 9 | 41.5 (32–50) | 9 | 34.5 (25–44) | 2 | 29 (14–44) | [0.590] | [0.611] | [*1.215*] | [*1.704*] | * Control > ALsci/ALSbi $p = 0.004$ |
| Delayed recall (Rey list) | 39 9.7 (9–11) | 11 | 8.5 (7–10) | 9 | 9.1 (6–12) | 8 | 7.3 (4–11) | 2 | 5.5 (0–11) | [0.362] | [0.190] | [0.753] | [*1.126*] | N.S. |
| Missing items (Rey List) | 39 0.7 (0–1) | 11 | 1.6 (0–3) | 9 | 1.2 (0–2) | 8 | 1.3 (0–3) | 2 | 3 (0–6) | [−0.603] | [−0.380] | [−0.499] | [*−1.519*] | N.S. |
| False hits (Rey list) | 39 0.9 (0–1) | 11 | 1.3 (0–3) | 9 | 0.9 (0–2) | 8 | 2.2 (0–5) | 2 | 9.5 (1–18) | [−0.175] | [0.037] | [−0.652] | [*−3.366*] | N.S. |
| *Language* | | | | | | | | | | | | | | |
| % Object naming (BADA) | 20 96.0 (94–98) | 9 | 94.4 (91–98) | 9 | 96.2 (93–99) | 8 | 90.4 (84–97) | 2 | 86.5 (83–90) | [0.306] | [−0.063] | [*0.939*] | [*1.973*] | N.S. |
| % Action naming (BADA) | 20 96.0 (94-98) | 9 | 92.0 (88-96) | 9 | 93.2 (84-100) | 8 | 85.7 (73-98) | 2 | 85.7 (82-89) | [0.763] | [0.360] | [*1.158*] | [*2.031*] | * Control > ALsci/ALSbi $p = 0.014$<br>uALS > ALsci/ALSbi $p = 0.050$ |

Data is given as group means. Number of measurements (n.), and 95% confidence intervals (C.I.) are reported. Hodges' g effect size is reported in squared brackets (significant high effect size are reported in bold). The significant p-values of post hoc comparisons (Mann-Whitney U test) are reported. * = Bonferroni correction for multiple comparisons ($p < 0.017$); N.S. = no significant difference. Legend: BADA = Battery for the Analysis of the Aphasic Deficit; CET = Cognitive Estimation Test; MMSE = Mini Mental State Examination; uALS = unimpaired ALS group; WCST = Wisconsin Card Sorting Test.

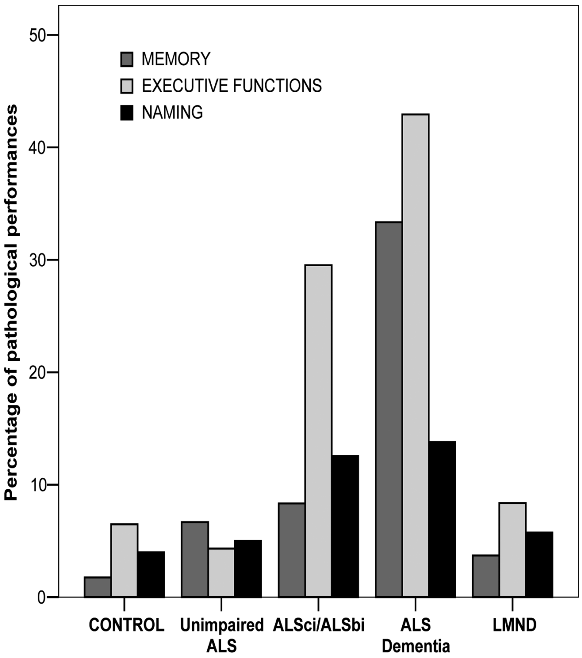

Fig. 2. The distribution of the mean number of pathological performances in memory, executive function and language domains. Legend: ALSci/ALSbi = cognitively and behaviourally impaired ALS patients; ALS Dementia = demented ALS patients; Control = Healthy volunteers; LMND = Lower Motor Neuron Disease patients; Unimpaired ALS = Cognitively and behaviourally normal ALS patients.

ALSci/ALSbi, demented ALS and LMND groups. Likewise, there were no differences in disease onset type, ALS-N, ALSSS spinal and bulbar scores. LMND patients had a longer disease history than unimpaired ALS ($U = 18.5$, $p = 0.006$), ALSci/ALSbi ($U = 18.5$, $p = 0.018$) and ALS/Dementia ($U = 4.0$, $p = 0.052$) groups.

On the basis of HDRS, none of the participants had severe depression and no differences were found between groups with regard to HDRS scores.

### 3.3. Neuropsychological findings

As for the mean percentage of pathological neuropsychological scores, we found that cognitively normal ALS, ALSci/ALSbi, LMND and control groups differed in the domains of executive function ($X_3 = 27.185$, $p < 0.001$), and language ($X_3 = 9.136$, $p = 0.028$). Figure 2 shows the mean percentage of pathological scores within cognitive domains and subject groups.

Post hoc comparisons, which survived the Bonferroni correction ($p < 0.016$), revealed that the ALSci/ALSbi group had a higher percentage of pathological scores in the executive function domain with respect to LMND ($p = 0.003$), healthy controls ($p < 0.001$) and cognitively-normal ALS ($p = 0.001$); and in the language domain with respect to healthy controls ($p = 0.013$) and cognitively-normal ALS ($p = 0.014$). The LMND, cognitively-normal ALS and control groups had comparable results. In contrast, ALS patients with dementia syndrome had more severe memory and executive dysfunction impairment than controls, cognitively-normal ALS and LMND groups.

Considering the neuropsychological test scores (Table 2), we found that the group of cognitively and behaviourally-normal ALS had comparable neuropsychological performances to healthy controls and LMND patients.

The impaired ALS patients (ALSci/ALSbi) had significantly inferior performances in most executive function tests, and also on digit span forward, short-term recall of the Rey list and action naming.

The LMND patients had control-like performances except for the digit span forward test ($U = 53$, $p = 0.023$) and the letter fluency index ($U = 75$, $p = 0.040$).

### 3.4. Behavioural findings

Behavioural data were available from 21 ALS patients and 9 LMND patients. Four out of 21 ALS patients (19%) had behavioural anomalies: 2 ALS patients fulfilled criteria for the behavioural variant of FTLD and 2 ALS cases satisfied criteria for behavioural impairment (ALSbi) [9]. The ALSbi patients were both 61 year old females. Both were described as disorganized and sporadically careless in their everyday behaviour.

Based on caregiver interviews, none of the LMND patients satisfied criteria for behavioural impairment, with the exception of a 60 year old woman who was described by clinicians as fatuous, perseverative and mentally rigid.

In order to identify the pattern of behavioural abnormalities in non-demented ALS patients, group comparisons were made using FBI reports obtained from cognitively normal ALS (10 cases), ALSci/ALSbi (8 cases) and LMND (9 cases). The FBI global score did not differ between groups ($X_2 = 3.653$, $p = 0.161$). No severe changes were observed in non demented patients, although mild to moderate changes in affect and social behaviour were reported in 5 unimpaired ALS patients (50%), 8 cognitively and/or behaviourally impaired ALS patients (100%) and 6 LMND patients

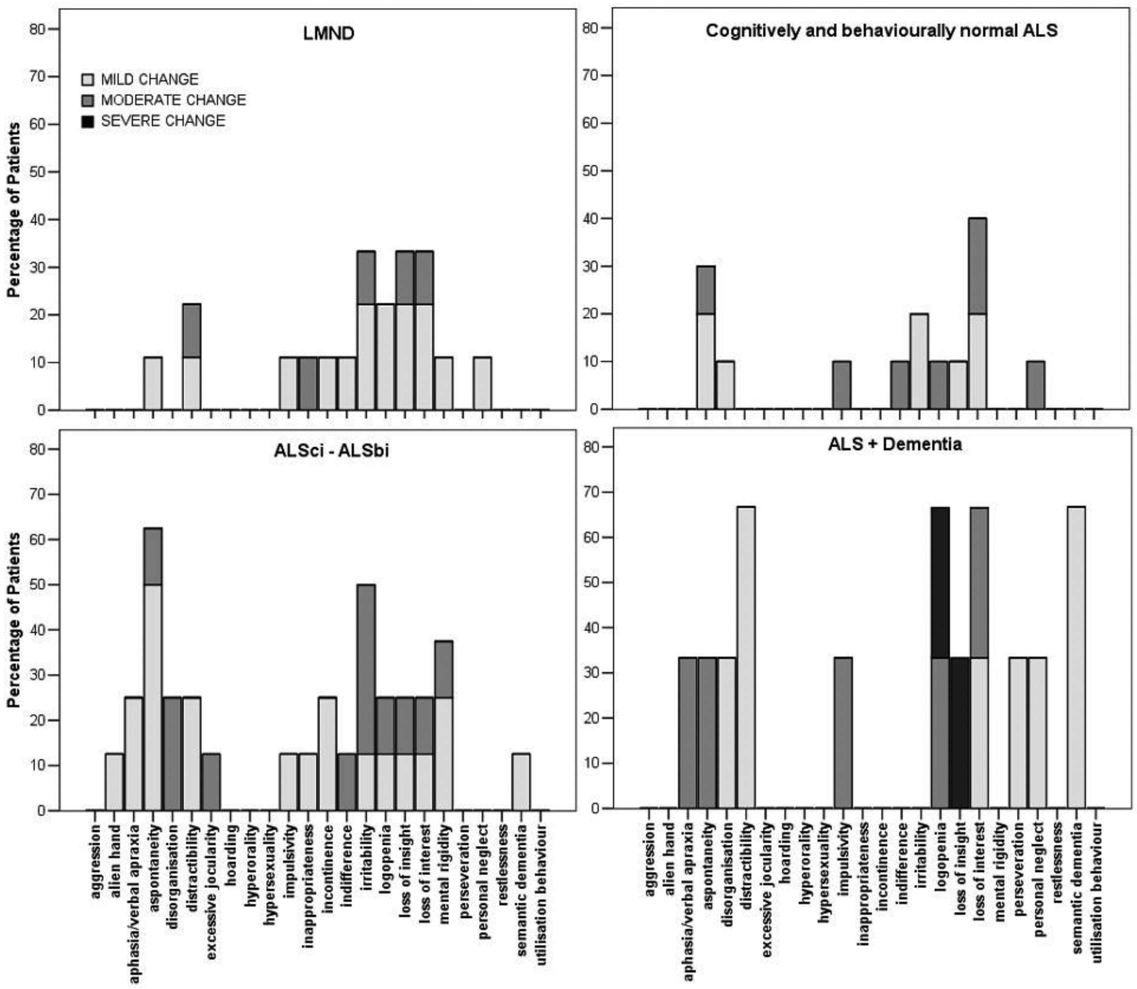

Fig. 3. Percentage of patients displaying mild, moderate and severe affective and social symptoms. Legend: ALSci − ALSbi = cognitively and behaviourally impaired ALS patients; ALS + Dementia = demented ALS patients; LMND = Lower Motor Neuron Disease patients.

(66%). ALSci/ALSbi and unimpaired ALS groups differed with respect to the prevalence of behavioural change ($p = 0.035$). Considering the FBI items separately, aspontaneity was more represented in cognitively/behaviourally impaired ALS (62%) with respect to the LMND group (11%, $p = 0.042$). Mental rigidity was slightly more represented in the ALSci/ALSbi group (37%) with respect to the unimpaired ALS group (0%, $p = 0.068$). See Fig. 3 for further details.

### 3.5. Correlational findings

In all ALS patients, cognitive performances did not correlate with disease severity or disease length. Also in the LMND group we did not find any significant correlation between clinical features of the disease and cognitive performances.

## 4. Discussion

This study assessed the cognitive and behavioural status of a sample of ALS patients without a previous diagnosis of dementia, according to the recent consensus criteria [9], verifying, for the first time, the impact of physical disability on cognitive and behavioural abnormalities by means of a LMND comparison group.

Consistent with the literature [3], we found a spectrum of frontal lobe dysfunctions in half of ALS patients. The cognitive and behavioural disturbances varied in severity, with 8 (36%) patients having subtle cognitive disturbances (ALSci), 1 (4%) demonstrating subtle behavioural impairments (ALSbi), 1 (4%) having both cognitive and behavioural anomalies and 3 (13%) fulfilling the diagnostic criteria for dementia (2 probable FTD and 1 probable AD) (Fig. 1).

The current study showed that ALSci patients had a similar pattern of cognitive and behavioural dysfunction as in the patients affected by ALS with dementia, but to a milder degree. In particular, demented ALS patients suffered from a general cognitive decline, involving executive function and memory domains, whereas cognitively and behaviourally impaired ALS patients displayed a relatively selective deficit in the executive function domain (Table 2, Fig. 2). This confirms previous studies highlighting that executive dysfunction is the main cognitive change linking ALS with FTD [6,12, 33,38,39]. Furthermore, we observed that behavioural disturbances in ALS were in general subtle and difficult to identify [40]. When they were specifically assessed, however, mild or moderate changes in affect and social behaviour were noticed. In our sample, the behavioural impairment in ALS patients with FTD and ALSbi was more severe than in unimpaired ALS patients (Fig. 3). This may be consistent with the notion of a continuum between ALS and FTD.

Our results also provides evidence that it is not possible to predict the development of cognitive and behavioural impairment in ALS on the basis of clinical or demographic data. Indeed unimpaired and impaired ALS patients were similar in age, education, bulbar and spinal motor symptoms.

Despite comparable demographic and clinical features, the ALS subgroups differed in the pattern of cognitive performance, as evidenced by the extensive neuropsychological evaluation used here. Compared to the cognitively-normal ALS patients, the ALSci/ALSbi group had deficits in verbal fluency (even when a measure to control for speed of response was used), and showed limited cognitive flexibility as assessed with the WCST and the CET. These findings are in agreement with the literature. Verbal fluency is considered a sensitive indicator of damage to frontal and temporal areas [41] and it is particularly sensitive to impairment in ALS [2,33,42]. Moreover, the ALSci/ALSbi patients' low performance at WCST can be considered to reflect frontal lobe dysfunction [43], with the perseverative errors as a clinically distinctive feature of dysexecutive syndrome [44].

Furthermore, the ALSci/ALSbi group was impaired in the action naming task in comparison to cognitively-normal ALS patients. This may be consistent with central semantic deficits of verb processing in ALS with dementia/aphasic syndrome [45,46]. In ALS, an interesting anatomical association between impaired ability to process actions in language and atrophy in the motor and premotor areas has been established [46,47]. An alternative possibility, as revealed by studies on FTD [48], is that executive difficulties might affect verb processing, as suggested by the association between action naming disorder and executive dysfunction.

With regard to behaviour, we documented that changes towards mental rigidity were found only in the ALSci group (Fig. 3). This suggests that inflexibility and mental rigidity might be considered as the behavioural counterpart of executive dysfunction.

In order to understand if such deficits are intrinsic to the ALS pathological process or rather due to physical limitations, we enrolled a group of LMND patients who had similar motor dysfunction (Table 1). Since some aspects of cognitive impairment can be exacerbated by motor disabilities [33], we aimed to identify those that are typical of ALS by means of group comparisons. In addition, to control for the impact of motor impairments on cognitive assessment, we selected neuropsychological tests with limited requirements for speech or manual abilities (e.g.: Raven coloured Matrices, WCST, CET), or tests taking into consideration individual variations in motor speed (e.g.: fluency indexes and the Stroop test measure).

As expected, we found that the cognitive status of LMND patients was comparable to healthy controls (Fig. 2), with the exception of verbal short-term memory (Table 2). Comparing ALS and LMND, we found that the number of patients with cognitive impairment was higher in ALS than LMND. More specifically, LMND and unimpaired ALS patients displayed similar cognitive performance, whereas the ALSci/ALSbi group had more severe executive dysfunction than the LMND group (Fig. 2) and showed impairment in the cognitive estimation task (CET) (Table 2). This supports the notion that executive dysfunction affects 50 per cent of ALS patients and is not exacerbated by physical disability, suggesting that it is due to a progressive cortical degeneration involving the frontal lobes.

Moreover, we suggest that the CET might be of clinical relevance in ALS, because it assesses the integrity of the executive system independent of motor disability, and because it investigates important functions of daily life, since many activities depend on guesses and estimates.

Other than executive dysfunction, ALSci patients also had deficits in verbal short-term memory, indicated by their lower scores in the digit span test and in the short-term recall of the Rey list compared to healthy controls (Table 2). A possible contribution of articulatory impairment interfering with the rehearsal process is suggested by the observation that also the LMND

group had a lower performance than the control group. It must be underlined that other studies have reported disorders of both working memory and long-term episodic memory not only in ALS, but also in patients with PMA [33]. In the present series one PMA patient had both executive dysfunction and behavioural impairment, supporting the possible existence of this new phenotype of motor neuron disease.

Our study also contributed towards establishing whether the behavioural impairments of ALS were related to the presence of FTD or to emotional reactions due to physical impairments [10,14]. Indeed, there is no doubt that receiving the diagnosis of a motor neuron disease has a substantial emotional impact on patients [49]. Also motor dysfunctions *per se* can impact on behaviour: patient's speech difficulties and dysarthria may have generated the reduction in social communication [40]. We disentangled this issue by considering the neurobehavioral features that were common to ALS and LMND groups as a reaction to illness.

The loss of interest in social and everyday activities could be explained as a reactive change, since we noticed it in ∼30% of ALS and LMND patients. A similar argument could be made for irritability (Fig. 3). Even if high levels of social irritability could be considered traits that distinguish ALSci/ALSbi patients from cognitively and behaviourally intact ALS patients [12], we agree with Gibbson and co-workers [10] suggesting that it has a low diagnostic specificity. Not all the behavioural changes we reported, however, could be explained as reactive. The high prevalence (62%) of aspontaneity in ALSci/ALSbi, compared to LMND, suggests that the impairment of volition may represent a behavioural symptom of frontal lobe damage. Disorganization may complete the neurobehavioral profile of ALS. Indeed, both ALSbi cases were reported as impaired in complex action planning. In view of the small sample size and the lack of available information about the premorbid state of patients [14], however, it is not possible to reach definitive conclusions about the behavioural profile of ALS.

In summary, impaired and unimpaired ALS patients had similar clinical features but differed in cognitive performances (verbal fluency, cognitive flexibility, action naming) and in the amount of behavioural anomalies. The ALS patients who were classified as cognitively and behaviourally impaired [9] (∼ 40%) manifested a relatively selective deficit in the executive function domain and behavioural changes, in particular apathy and disorganization. We demonstrated that their impairment was not influenced by physical limitations.

A major limitation of the study is the small number of patients. So far, it has not been possible to establish if ALSci/ALSbi constitutes a pre-morbid state of dementia in ALS. A larger sample size and follow up evaluations are needed to verify if patients with diagnosis of both cognitive and behavioural anomalies progress to FTD or other dementia syndromes, or of they represent a separate phenotype of ALS, non necessarily progressing to dementia.

## Conflict of interest

None.

## Acknowledgments

We wish thank our colleagues of the Neurology and Neurorehabilitation Unit of San Raffaele Turro Hospital, particularly: Dr. Alessandra Marcone, Dr. Luca Bernasconi, Dr. Michele Zamboni, Dr. Valeria Golzi, Dr. Maria Cristina Giusti for the clinical assessment of patients, Dr. Valentina Plebani and Dr. Elena Farini for their help in neuropsychological assessments. We are grateful to Henrietta Howells for the review of the manuscript and to Dr. Stefania Rossi for her help in recruiting and testing controls. A special thank to all the patients and volunteers who participated in the current study. The study was supported by a Ministry of Health grant (RF07-ALS).
